# ICU Patients’ Perception of Sleep and Modifiable versus Non-Modifiable Factors That Affect It: A Prospective Observational Study

**DOI:** 10.3390/jcm11133725

**Published:** 2022-06-28

**Authors:** F. Eduardo Martinez, Amber-Louise Poulter, Charuni Seneviratne, Abbey Chrimes, Kenneth Havill, Zsolt J. Balogh, Gemma M. Paech

**Affiliations:** 1 Department of Critical Care Services, John Hunter Hospital, Newcastle, NSW 2305, Australia; ed.martinez@health.nsw.gov.au (F.E.M.); amberlouise.poulter@health.nsw.gov.au (A.-L.P.); charu.seneviratne@gmail.com (C.S.); abbey.chrimes@health.nsw.gov.au (A.C.); ken.havill@health.nsw.gov.au (K.H.); 2 School of Medicine and Public Health, University of Newcastle, Newcastle, NSW 2308, Australia; gemma.paech@health.nsw.gov.au; 3 Department of Traumatology, John Hunter Hospital, Newcastle, NSW 2305, Australia; 4 Department of Respiratory and Sleep Medicine, John Hunter Hospital, Newcastle, NSW 2305, Australia

**Keywords:** critical care, hospitalization, sleep quality, circadian rhythm, environment

## Abstract

*Background*: Good sleep quantity and quality are essential for patient recovery while in the intensive care unit (ICU). Patients commonly report poor sleep while in the ICU, and therefore, identifying the modifiable factors that patients perceive as impacting their sleep is important to improve sleep and recovery. This study also assessed night-time light and sound levels in an ICU in an effort to find modifiable factors. *Methods*: A total of 137 patients (51F) aged 58.1 ± 16.8 years completed a survey including questions about their sleep before and during their ICU stay, factors contributing to poor sleep in the ICU, and perceived factors that may have improved their sleep in the ICU. Night-time light and sound levels were measured in patient rooms and nurses’ stations. *Results*: Patients reported poorer sleep quantity and quality while in the ICU compared to home. Among the most common reasons for poor sleep, easily modifiable factors included noise (50.4%) and lights (45.3%), potentially modifiable factors included pain (46.7%), and non-modifiable factors included IV lines (42.3%). Patients felt their sleep would have been improved with interventions such as dimming lights (58.4%) and closing doors/blinds at night (42.3%), as well as potentially implementable interventions such as a sleeping pill (51.8%). Overnight sound levels in bedrooms were above the recommended levels (40 dB) and light levels averaged over 100 lux. *Conclusions*: Sleep quality and quantity were both worse in ICU than at home. Modifiable factors such as sound and light are common factors that patients perceive impact their sleep in the ICU. Readily implementable sleep management strategies aimed at minimizing the impacts of sound and light levels in the ICU are ways to improve patients’ sleep in the ICU.

## 1. Introduction

Sleep is vital for overall health and wellbeing [1]. The critical care environment is unfavorable for achieving good quality and quantity of sleep [2]. Poor sleep is one of the most common complaints from patients during their intensive care stay, along with pain, thirst, and fear and anxiety [3,4]. It is recognized that patients in intensive care units (ICU) have disturbed sleep. Sleep latencies and arousals are increased, while sleep efficiency, slow-wave sleep (SWS), and rapid eye movement (REM) sleep are decreased [5,6].

The negative impact of sleep loss on healthy individuals is widely reported [7]. In ICU, sleep loss has additional implications for patients’ health and recovery, such as disturbances to ventilatory, cardiovascular, and immunological functioning, along with an increased risk of developing delirium and longer-term decreased quality of life [6,8]. Improving sleep quantity and quality in hospitals and in ICU is important to improve patients’ recovery from illness and injury. Further, interventions aimed at improving patients’ sleep can be easily targeted with cheap and effective and potentially non-pharmacological interventions.

The primary causes of poor sleep in the ICU reported by patients are noise, pain, light, loud talking, and intravenous catheters [9,10]. Noise levels in the ICU are commonly higher than those recommended by the World Health Organization (WHO, Geneva, Switzerland) [11,12,13,14,15], which suggests that sound should not exceed 30 decibels (dB) and maximum individual sounds should be less than 40 dB during the night [16].

Light levels in the ICU can also be discordant with the natural light–dark cycle [14,17,18], which is significant due to the direct impact of light on the timing of the sleep–wake cycle [19].

Sources of night-time noise and light in ICU are varied. Some night-time noise is due to factors that are unavoidable and unmodifiable, such as life-saving procedures (such as endotracheal intubations or resuscitations during a cardiac arrest), new admissions arriving to ICU (from operating theatre or emergency departments), or patients being transported to other locations in the hospitals (such as radiology or operating theatres). Further, bright lights might be unavoidable when medical procedures are being undertaken, when patients are being reviewed by medical staff, or when nursing care is being delivered. On the other hand, some noise and light can be more modifiable, such as dimming lights inside and outside of patients’ rooms, lowering the volume of alarms and devices in use, and limiting the number of people allowed to visit the ICU at night.

Despite evidence demonstrating potential causes for poor sleep in the ICU, few studies have investigated what factors patients themselves feel could be modified to improve sleep. It is unknown whether the noise and light levels in the ICU that are perceived by patients as being detrimental to sleep are factors that can be modified or if these are unavoidable noises and lights due to ICU-specific factors such as procedures, resuscitations, new admissions, and discharges. Therefore, the aim of the current study was to assess patients’ perceptions of quality and quantity of sleep in the ICU, patients’ perceptions of the sources of noise and light, and to determine the actual sound and light levels in the ICU. It was hypothesized that patients would report poor sleep and that this poor sleep would be due to predominately modifiable factors around noise and light.

## 2. Materials and Methods

### 2.1. Participants

All patients admitted to the ICU were screened to assess eligibility. Patients were eligible to participate if they: (1) were aged 18 years or older; (2) had a minimum of two nights ICU stay; and (3) had adequate cognitive ability to answer a questionnaire. Cognitive ability was assessed by ruling out the effect of sedative agents and with a series of questions about time, date, location, and recent current events. Patients were excluded if they: (1) were admitted to the ICU after cardiothoracic surgery, as these patients are admitted to the ICU on invasive mechanical ventilation, have a short ICU day, and the usual practice is for extubation during the evening of their admission day; (2) were expected to die during ICU admission; or (3) had any congenital or acquired neurological deficits that may prevent them from answering the questionnaire appropriately.

The study was approved by the Hunter New England Human Research Ethics Committee (HREC/16/HNE/343) according to guidelines established by the National Health and Medical Research Council of Australia. Patients provided written informed consent before participating.

### 2.2. Setting

This was an observational, prospective, single-center study in the ICU of John Hunter Hospital, a tertiary-referral hospital in Newcastle, New South Wales, Australia. The ICU is a medical and surgical unit that provides services to a range of departments, including trauma, neurosurgery, cardiothoracics, and interventional radiology. It provides comprehensive organ support, which includes outreach services, 24-hour echocardiography, and extracorporeal life therapies, including extracorporeal membrane oxygenation.

The ICU consists of 29 physical rooms. It has a functional capacity for up to 26 patients, so three rooms remain vacant at any given time. These rooms are spread across five zones, with one nurses’ station in each zone. Zone A and Zone B are the two main zones, with 8 rooms each. The remaining three zones, Zone C, Zone D, and Zone E, are smaller and have between 4 and 5 rooms each.

### 2.3. Questionnaire

ICU patients who met screening criteria and provided written informed consent were invited to complete the questionnaire after being discharged from ICU, but while they were still in hospital. The questionnaire was administered by the research team, with patients being asked the questions by the researchers and the answers being logged into a secure online platform by the researchers. The survey was based on a previously developed questionnaire by Little et al. [9]. Patients were assigned a de-identified study number, and the answers provided were stored by the person administering the questions in a secure, password-protected, pay-per-use, online platform (SurveyMonkey Inc. San Mateo, CA, USA, www.surveymonkey.com, last accessed on 26 November 2019).

The questionnaire asked about general patient demographics, including age and sex and time of day of completion. Patients were asked about their overall sleep quality and quantity at home and while in the ICU. The questionnaire asked patients about their sleep/wake cycle and whether they obtained enough sleep while in the ICU. Patients were asked to select the factors they felt contributed to poor sleep in the ICU from a list and the factors that they believed would have improved their sleep in the ICU from a second list.

### 2.4. Sound and Light

Overnight sound and light levels were measured between 22:00 h and 06:00 h. Samples were taken from both patient rooms and nurses’ stations, including rooms with and without windows. To obtain the best representation possible, rooms and nurses’ stations having sound and light levels measured were rotated, with a different room and station selected each study night. Despite this, some rooms had more recordings than others. Reasons included the room not being in use, procedures with multiple people being undertaken in the room, or when patients were receiving end-of-life care. Measuring noise and light levels in rooms that were not being used meant that there would be no noise and light coming from the room; therefore, everything measured would be coming from the outside only and could give us information about how much noise and light is being filtered in from the outside. Best attempts were made to ensure the positioning and recording of light and sound were consistent each time they were measured.

Nine of the rooms have no windows or natural light so that patients cared for in these rooms were not exposed to natural sunlight. Only rooms in the two main 8-bed pods and their respective nurses’ stations (Zone A and Zone B) were subject to sound and light measurements, as these are the main zones.

Sound levels (dB) were taken using a Standard ST8852 Pro Sound Level Meter datalogger (Springboro, OH, USA). Sound measurements were taken every 2 s with the “continuous measurements” feature on the sound level monitor from a fixed location on a shelf in the room or nurses’ station. Only the study team, who had experience with the measuring devices, was responsible for setting up the instruments to collect sound levels in both the rooms and nurses’s stations. Sound level data was later downloaded from the sound level meter to a computer for it to be analyzed.

Overnight light levels (lux) were taken every hour using a Sper Scientific’s LED light meter 850006 (Scottsdale, AZ, USA) by nursing staff. Senior ICU nursing staff were provided with information about the light measuring devices and were shown by the study team how to adequately calibrate and then collect measurements with the light level monitor. Nurses were asked to take measurements with the study team present to assess if they were utilizing the instruments correctly before being asked to collect data. The participating nurses allocated to being the Clinical Support Nurse for the shift were instructed to quietly and without disturbing the patient enter the room to take a static light measurement in the same spot each hour. They would then take a static measurement from the same spot of the corresponding nurses’ station for the room being measured.

### 2.5. Statistical Analysis

All data were analyzed using SPSS v.28 for Windows. Separate mixed model ANOVAs were performed to assess for main and interaction effects of ‘time’ and either ‘room’ (patient rooms) or ‘zone’ (nurses’ stations) for overnight light levels. Separate mixed model ANOVAs were also performed to assess for the main effects of ‘time’ for overnight sound levels in patient rooms and nurses’ stations. Where significant main effects were found, post hoc analyses using pairwise comparisons (Bonferroni) were performed. As light measurements were not normally disturbed, data were log transformed prior to statistical analyses. Data are presented as mean ± SD unless otherwise stated. Significance was assumed at *p* ≤ 0.05.

## 3. Results

### 3.1. Questionnaire

A total of 141 patients answered the questionnaire. Results from four patients were excluded due to incomplete questionnaire responses (less than 10% completed). Of these remaining 137 patients, the average age was 58.0 ± 16.8 years, and 63% (87/137) were male. The most common sources of admission were Operating Theatre with 39.4%, which included emergency surgery patients, followed by the Emergency Department with 37.2%, which included major trauma patients. The average Acute Physiology and Chronic Health Evaluation-III (APACHE-III) score for the cohort was 59 ± 29. The most common diagnoses on admission to ICU were “Septic shock” with 11% (15/137), “Respiratory, medical” with 10% (14/137), and “Trauma“ with 10% (13/137). Of all the patients who answered the survey, 43% (59/137) had been invasively mechanically ventilated at some point during their admission.

**Figure 1:**
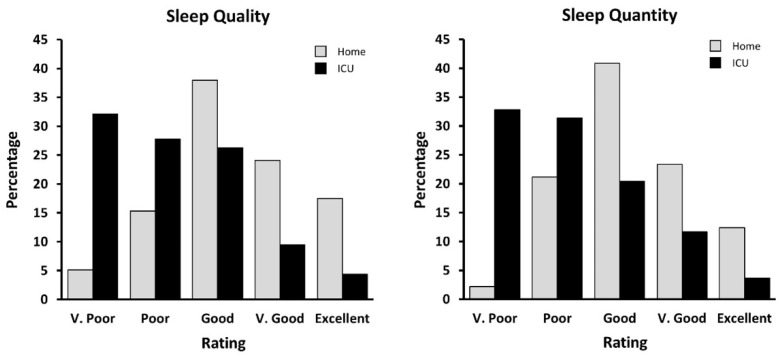
Self-reported sleep quality (**left**) and quantity (**right**) at home (grey) and while in the ICU (black).

Most patients reported experiencing “good” quality (38.0%) and “good” quantity (40.9%) of sleep at home, but most reported “very poor” quality (32.1%) and “very poor” quantity (32.9%) while in the ICU (Figure 1). More than half (55.5%) of patients reported not having a normal sleep-wake cycle in the ICU. Only 37.2% felt they obtained enough sleep, with 59.9% reporting not getting enough sleep and 2.9% being unsure. Of those that reported obtaining enough sleep, most (76.5%) reported their sleep as being refreshing. (Figure 1).

Factors that patients felt impacted their sleep were categorized as either easily modifiable (i.e., could be easily changed), potentially modifiable (i.e., could be changed, but with significant alterations to ICU environment and processes), or non-modifiable (i.e., mostly things that are out of the control of ICU staff and administrators).

When patients were asked about the factors that led to experiencing poorer sleep in the ICU, the factors ranked highest were mostly easily modifiable, such as noise and light, or potentially modifiable, such as pain and thirst (Table 1). The lowest ranking factors were mostly non-modifiable, such as sleep disorders and breathing machine, among others (Table 1). It was possible for patients to select more than one answer. There were 33.6% (46/137) of patients who selected three or fewer options, 24.8% (34/137) of patients who selected four or five options, and 41.6% (57/137) of patients who selected more than five answers. The frequency with which factors were mentioned by respondents based on their gender was similar (Supplementary Table S1).

Factors that patients felt could improve their sleep in the ICU were environmental, pharmacological, and those associated with ICU processes and ways of doing things. They were categorized as easy to implement and potentially implementable. When patients were asked about factors that they felt could have improved their quality and quantity of sleep while in ICU, the most reported factors were either environmental or pharmacological factors. Over half of the patients reported that dimmed lights and a sleeping pill would have helped their sleep (Table 2). It was possible for patients to select more than one answer for this question as well. There were 30.7% (42/137) of patients who selected three or fewer options, 24.1% (34/137) of patients who selected four or five options, and 45.3% (62/137) of patients who selected more than five answers. The frequency with which factors were mentioned by respondents based on their gender did not seem to differ (Supplementary Table S2).

### 3.2. Sound

Sound recordings were taken across 16 nights in patient rooms totaling 207 measurements, and across 22 nights in nurses’ stations totalling 283 measurements. Measurements taken in patients’ rooms were done when there was a patient on invasive mechanical ventilation on 38% (6/16) of the nights. Overnight sound levels averaged 52.8 ± 3.6 dB in patient rooms and 53.8 ± 3.8 dB in nurses’ stations.

**Figure 2:**
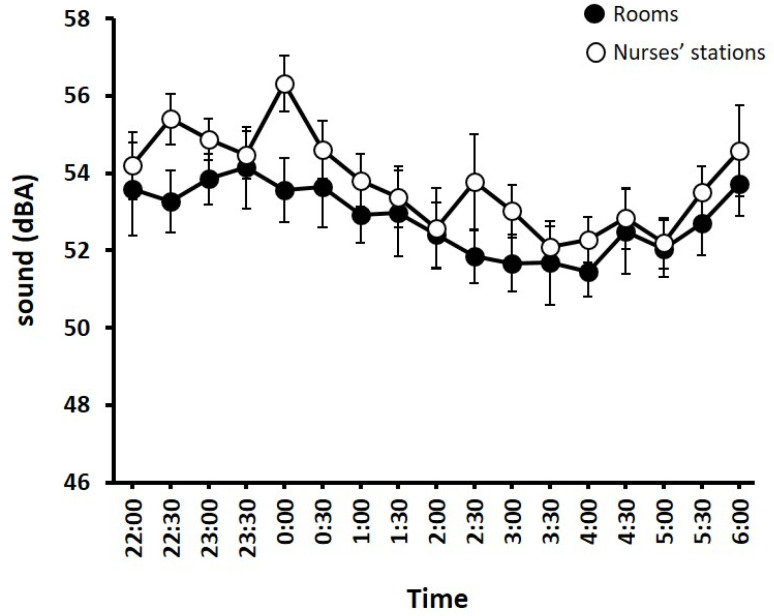
Sound levels (dB) in rooms (closed circles) and nurses’ stations (open circles) overnight. Error bars are SEM.

For patient rooms, there was no main effect of ‘time’ (F _(16,254)_ = 0.94, *p* = 0.52), such that sound was constant across the night. For the nurses’ stations, there was a main effect of ‘time’ (F _(16,354)_ =2.32, *p* < 0.05). Pairwise comparisons showed that nurses’ stations were louder (*p* < 0.05) at midnight (56.3 ± 3.4 dB) compared to 03:30 (52.1 ± 2.4 dB), 04:00 (52.3 ± 2.8 dB), and 05:00 (52.2 ± 3.0 dB) (Figure 2).

### 3.3. Light

Light levels were taken over 18 nights from 10 different patient rooms totalling 146 measurements and over 15 nights from 2 different nurses’ stations (Zones A and B) totaling 134 measurements. Measurements taken in patients’ rooms were done when there was a patient on invasive mechanical ventilation on 39% (7/18) of the nights. Overnight light levels averaged 104.1 ± 203.6 lux in patient rooms and 495.5 ± 367.4 lux in nurses’ stations.

**Figure 3:**
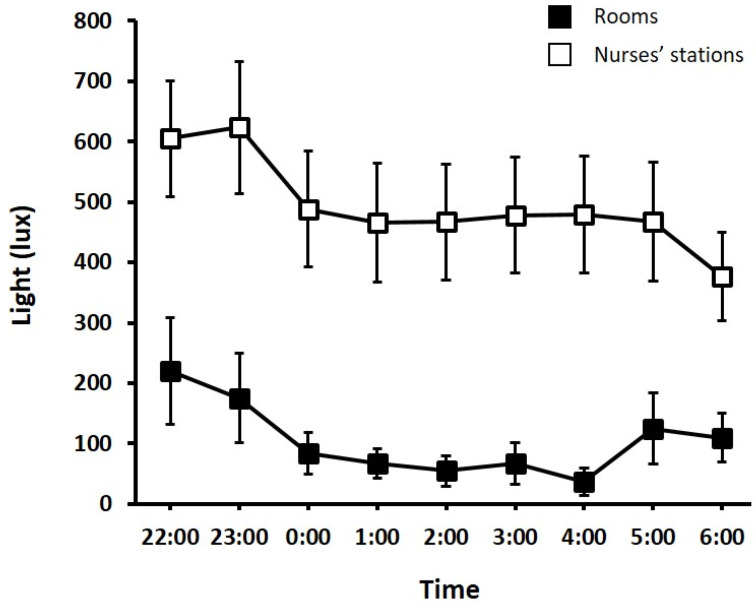
Light levels (lux) in rooms (closed squares) and nurses’ stations (open squares) overnight. Error bars are SEM.

For patient rooms, there was a main effect of ‘room’ (F _(9,56)_ =2.18, *p* < 0.05). On average, room number 2 (no window) was significantly (*p* = 0.03) brighter (386.1 ± 441.4 lux) than room number 12 (window) (11.7 ± 21.3 lux). There was no effect of ‘time’ (F _(8,56)_ = 0.75, *p* = 0.65), such that overnight light levels remained stable (Figure 3). There was also no interaction effect between ‘room’ and ‘time’ (F _(72,56)_ = 0.47, *p* = 1.00), suggesting that the differences in rooms were consistently different across all time points.

For nurses’ stations, there was a main effect of ‘zone’ (F _(1,116)_ =5.06 *p* < 0.05), with Zone A being significantly darker on average (492.5 ± 332.0 lux) than Zone B (500.1 ± 419.0 lux). There was no effect of ‘time’ (F _(8,116)_ = 0.52, *p* = 0.84), with light levels remaining consistent across the night (Figure 3). There was also no interaction between ‘room’ and ‘zone’ (F _(8,116)_ = 0.11, *p* = 1.00), suggesting that the differences in light levels between stations were consistent at all time points.

## 4. Discussion

Due to the nature of the ICU environment, it is easy to see how this is an unfavorable environment for patients to achieve good sleep. However, understanding the factors that make the ICU an unfavorable sleeping environment, particularly from the patient’s perspective, can allow for improvements to the environment being made and therefore help to improve sleep and recovery.

In line with previous studies [5,9,20,21], patients reported that their sleep quality and quantity were poorer while in the ICU compared to their sleep at home. The poor sleep while in the ICU is of concern to medical staff and patients due to the vital role that sleep plays [1,22], including in the recovery process during and following critical illness.

Improving sleep in ICU likely requires that the problem be tackled from many angles. In this study, we classified factors that patients reported as impacting sleep into easily modifiable, potentially modifiable, and non-modifiable. In this way, ICUs can focus and invest time and effort in addressing the factors that can be easily modifiable first, followed by those potentially modifiable, and at least consider the ones that we have deemed non-modifiable.

In the current study, and in line with previous reports [9,23], the most common factor that patients reported as impacting their sleep was sound. While sound levels were higher than recommended levels [16], the levels observed here were similar to, and in some instances lower than, previous reports [12,14,18]. Sound levels in patients’ rooms on average (52.8 ± 3.6 dB) were slightly quieter than normal conversation, which is around 60 dB, suggesting that the night-time sound levels were not extremely high. This means that sound does not have to be excessively high for patients to feel that it affects their sleep. Importantly, current recommendations for noise levels in the ICU and hospitals are over 20 years old [16] and may not be reflective of modern ICU environments.

It is important to note that sound levels in patients’ rooms and nurses’ stations were the same, as evidenced by Figure 2. With this being the case, it means that either there was a lot of noise coming from inside the room or that the noise outside the room was carrying into the room very easily. This was despite the current study having been conducted in a unit containing single rooms with doors. We can assume that in ICU with open bays, noise carrying inside patient rooms from the outside would be just as much or even more. Based on current results relating to the factors patients felt would have improved their sleep, ICUs could easily implement routines to close doors and blinds at certain times of night to try and modify this noise impact on patients’ sleep. Such a routine could also incorporate lowering the volume of the ventilators and alarms inside the rooms to further reduce noise. Obviously, any lowering of ventilators and alarms in the room would need to be done in conjunction with strategies to alert nurses efficiently enough when those alarms are trying to warn of physiological derangements by assuring that the monitors are being observed from the nurses’ stations.

Also in concordance with previous reports [9], patients reported that lights (45.3%) impacted their sleep in the ICU. Overall, overnight light levels were relatively low inside patient rooms, quite often less than 100 lux, which is similar to outdoor light on a very overcast day. Despite light levels being lower inside the rooms than outside (i.e., in the nurses’ stations), the fact that patients identified light as affecting their sleep and suggested dimmed lights meant that the light outside was likely filtering inside the room. It may also be possible that patients were disturbed by lights being turned on inside the room at night and/or by lights from machines. Further, of all the factors that patients felt would improve their sleep in the ICU, dimmed lights (58.4%) were the most common factor.

In the ICU, even what may be low or short intensity light levels could lead to circadian misalignment and poorer sleep. Shielding patients’ eyes when bright light is required, such as when doing procedures, or having patients wear eye masks at night could be strategies to minimize light exposure at night. Indeed, almost a quarter of patients (24.8%) reported that using eye masks would likely improve their sleep.

It is important to note that there was a lot of variability in overnight light levels in patients’ rooms. The maximum lighting level recorded was 1212 lux, similar to outdoor light levels on an overcast day, and the minimum was 0.2 lux, similar to outdoor light at night with a full moon. This variability may be due to how light measurements were taken with possible differences between different nursing staff. However, it is unlikely that this explained all the variability, with bursts of bright light (e.g., lights being turned on in patient rooms) common in the ICU [18]. The impact of different types of light exposure on the circadian rhythm system, including low light (e.g., ~180 lux) and intermittent light, is well documented [24,25,26].

Importantly, neonatal and pediatric intensive care units use many interventions and night-time routines aimed at reducing noise and light at night with success. In these units, sleep management plans are routinely utilized where lights are dimmed or turned off at certain predetermined times. Noises from alarms, telephones, and conversations are kept to a minimum overnight. Nursing and medical interventions are rationalized during the night so that only the most urgent procedures are attended to, with everything else left for the daytime so that sleep is not interrupted. In adult ICUs, bundles of routine care are already widely used, which is the case with FAST-HUG, for instance [27]. These remind clinicians to pay attention to interventions that prevent complications and avoid morbidity. Easy to modify interventions aimed at improving patient-perceived quality and quantity of sleep, such as closing blinds, lowering the volume of alarms inside the rooms, or offering patients earplugs and eye masks to shield them from non-modifiable sources of light and sound, could be incorporated into these widely accepted bundles of care to make improving sleep a routine part of ICU practice. Currently, there is mixed evidence about the effectiveness of earplugs and eye masks in the ICU for improving patient sleep [28], but these have not been looked at when used as part of a bundle of care.

Interestingly, over half of the surveyed patients (51.8%) felt that having a sleeping pill would have improved their sleep in the ICU. While sleeping pills seem to be an easy fix to improve sleep in the ICU, pharmaceuticals such as benzodiazepines are generally only suggested as a short-term solution due to their contraindications, side effects, and modest efficacy [29], which can make these unsuitable for some patients. Melatonin has been suggested as an alternative pharmacological agent, as it is typically associated with fewer side effects and has less impact on sleep architecture. However, the effectiveness of melatonin in improving sleep in the ICU has not been clearly demonstrated [30]. There is now more robust evidence that melatonin does not help prevent complications associated with lack of sleep, such as delirium [31]. The use of pharmacological treatments in ICU to aid sleep, like with pain, is best done on a case-by-case basis.

While the current study offers insights into the patient’s experiences in the ICU, there are some limitations that need to be considered. Firstly, variations in how the staff took light measurements may explain some of the large variability observed in current lighting levels. Secondly, only night-time light levels were assessed, and the wavelength of light was not measured. Both daytime light levels and the wavelength of light can impact circadian timing [24,32], and therefore, future studies should consider including these additional measurements. Thirdly, while this study asked patients about what factors they felt could improve their sleep in the ICU, the efficacy of these factors has not been widely investigated in the ICU, and therefore, it is unclear if making the suggested changes would have any discernible impact on patients sleep in the ICU. Lastly, out of the vast number of sound level data points that were obtained, only the sound levels that were every 30 min were analyzed. This was conducted with the objective of keeping the number of data points manageable. It is possible that this approach might have influenced the results, but the authors felt that the results would still be representative.

## 5. Conclusions

The current study investigated patient perceptions of sleep in the ICU with particular regard to sound and light levels. Patients perceived that they had poorer sleep quantity and quality in the ICU. Noise and light were among the modifiable factors. Potentially modifiable factors included pain and feeling thirsty. Non-modifiable factors included IV lines and procedures.

Patients felt that readily implemented changes such as dimmed lighting, closing door/blinds at night, and personal pillow/keepsakes would have improved their sleep. Results highlight that there are interventions that could be incorporated into ICU care bundles that could potentially improve patients’ sleep and therefore recovery.

## Figures and Tables

**Table 1:** Self-reported factors that led to poor sleep in the ICU.

Factors	Count *n* = 137	%
**Modifiable**		
Noise (staff, alarms, TV)	69	50.4
Lights	62	45.3
Loud talking	52	38.0
Time disorientation	50	36.5
Temperature (too cold/hot)	41	29.9
Bed/pillow	40	29.2
Hungry	26	19.0
Visitors	18	13.1
Bathing	7	5.1
**Potentially Modifiable**		
Pain	64	46.7
Thirsty/dry mouth	61	44.5
Discomfort in position	43	31.4
People in room	34	24.8
Touch/move	28	20.4
Medication administration	27	19.7
Absence of partner	27	19.7
Tests/X-rays	25	18.2
Team rounding	24	17.5
Bed inflation and deflation	14	10.2
Restrained/confined	12	8.8
**Non-modifiable factors**		
IV lines	58	42.3
Anxiety	44	32.1
Procedures/measurements	42	30.7
Confusion	40	29.2
Nightmares/hallucinations	36	26.3
Tubes (nose/rectal/bladder)	29	21.2
Not being tired	24	17.5
Other patients	21	15.3
Suctioning	18	13.1
Endotracheal tube	17	12.4
Breathing machine	13	9.5
Sleep disorder	4	2.9

**Table 2:** Factors patients felt would improve their sleep in the ICU.

Factors	Count *n* = 137	%
**Easy to Implement**		
Dimmed light	80	58.4
Sleeping pill	71	51.8
Closing doors/blinds at night	58	42.3
Personal pillow/keepsake	49	35.8
Clock in the room	48	35.0
Ear plugs	43	31.4
Relaxation techniques	43	31.4
Eye mask/blindfolds	34	24.8
Music therapy	34	24.8
White noise	30	21.9
More blankets	28	20.4
**Potentially Implementable**		
Pain/more pain medication	48	35.0
Removal of monitors/alarms	47	34.3
No interruptions/unnecessary interruptions	41	29.9
Different bed	34	24.8
Window in room (if none)	16	11.7

## Data Availability

Data collected and presented here can be made available on direct request to the corresponding author.

## Supplementary Materials

The following are available online at https://www.mdpi.com/article/10.3390/jcm11133725/s1, Table S1: Factors contributing to poor sleep based on patient gender. Table S2: Factors patients felt could improve sleep based on patient gender.
