# Supplementary material for: ICU Patients’ Perception of Sleep and Modifiable versus Non-Modifiable Factors That Affect It: A Prospective Observational Study
Source: J Clin Med. 2022 Jun 28;11(13):3725. doi: 10.3390/jcm11133725 (PMC9267898; doi:10.3390/jcm11133725)
Supplement: Supplementary file 1 [file jcm-11-03725-s001.zip › jcm-1760294-supplementary.pdf]

Supplementary Material

Supplementary Table S1. Factors contributing to poor sleep based on patient gender.

	TOTAL		MALES		FEMALES	
Causes for poor sleep	Count	Percentage	Count	Percentage	Count	Percentage
Noise (staff, alarms, TV)	69	50.36	44	50.57	24	48.00
Pain	64	46.72	41	47.13	22	44.00
Lights	62.0	45.26	37.0	42.53	24.0	48.00
Thirsty/dry mouth	61	44.53	43	49.43	18	36.00
IV lines	58	42.34	31	35.63	26	52.00
Loud talking	52	37.96	32	36.78	20	40.00
Time disorientation	50	36.50	27	31.03	23	46.00
Anxiety	44	32.12	26	29.89	17	34.00
Discomfort in position	43	31.39	27	31.03	15	30.00
Procedures/measurements	42	30.66	26	29.89	16	32.00
Temperature (too cold/hot)	41	29.93	27	31.03	14	28.00
Confusion	40	29.20	23	26.44	15	30.00
Bed/pillow	40	29.20	25	28.74	15	30.00
Nightmares/hallucinations	36	26.28	22	25.29	14	28.00
People in room	34	24.82	20	22.99	13	26.00
Tubes (nose/rectal/bladder)	29	21.17	18	20.69	11	22.00
Touch/move you	28	20.44	17	19.54	11	22.00
Medication administration	27	19.71	19	21.84	8	16.00
Absence of partner	27	19.71	15	17.24	12	24.00
Hungry	26	18.98	15	17.24	11	22.00
Tests/x-rays	25	18.25	18	20.69	7	14.00
Not being tired	24	17.52	18	20.69	6	12.00
Team rounding	24	17.52	18	20.69	6	12.00
Other patients	21	15.33	13	14.94	8	16.00
Visitors	18	13.14	14	16.09	4	8.00
Suctioning	18	13.14	13	14.94	5	10.00
Endotracheal tube	17	12.41	13	14.94	4	8.00
Bed in/de-flation	14	10.22	6	6.90	8	16.00
Breathing machine	13	9.49	11	12.64	2	4.00
Restrained/confined	12	8.76	7	8.05	5	10.00
Bathing	7	5.11	4	4.60	3	6.00
Other (please specify)	5	3.65	2	2.30	3	6.00
Sleep disorder	4	2.92	2	2.30	2	4.00

Supplementary Table S2. Factors patients felt could improve sleep based on patient gender.

	TOTAL		MALES		FEMALES	
What would have improved sleep	Count	Percentage	Count	Percentage	Count	Percentage
Dimmed lights	80	58.39	50	57.47	28	56.00
Sleeping pill	71	51.82	44	50.57	26	52.00
Closing door/blinds at night	58	42.34	37	42.53	20	40.00
Personal pillow/keepsake	49	35.77	29	33.33	20	40.00
Pain medication	48	35.04	33	37.93	14	28.00
Clock in the room	48	35.04	29	33.33	19	38.00
Removal of monitors/alarms	47	34.31	29	33.33	17	34.00
Ear plugs	43	31.39	27	31.03	16	32.00
Relaxation techniques	43	31.39	27	31.03	16	32.00
No unnecessary interruptions	41	29.93	30	34.48	11	22.00
Eye mask/blindfold	34	24.82	23	26.44	11	22.00
Different bed	34	24.82	25	28.74	9	18.00
Music therapy	34	24.82	24	27.59	10	20.00
White noise	30	21.90	21	24.14	9	18.00
More blankets	28	20.44	18	20.69	10	20.00
More info about your health	20	14.60	11	12.64	9	18.00
Window in room (if none)	16	11.68	11	12.64	5	10.00
Other (please specify)	13	9.49	9	10.34	4	8.00
